# Exploring the Interplay Between Core Stability, Pulmonary Function, and Cardiorespiratory Fitness in Older Adults: A Randomized Controlled Trial of an 8-Week Mat Pilates Intervention

**DOI:** 10.3390/geriatrics11020043

**Published:** 2026-04-09

**Authors:** Bulin Jirapongsatorn, Decha Chinaksorn, Kanapot Pengked, Wannaporn Tongtako, Timothy Mickleborough

**Affiliations:** 1 Department of Sport and Exercise Science, School of Medicine, Walailak University, Nakhon Si Thammarat 80160, Thailand; decha.ch@wu.ac.th; 2 Center of Excellence in Tropical Pathobiology, Walailak University, Nakhon Si Thammarat 80160, Thailand; 3 Division of Sports Medicine, Department of Orthopedic Surgery, School of Medicine, Walailak University, Nakhon Si Thammarat 80160, Thailand; kanapot.pe@wu.ac.th; 4 Area of Exercise Physiology, Faculty of Sports Science, Chulalongkorn University, Bangkok 10330, Thailand; wannaporn.t@chula.ac.th; 5 Department of Kinesiology, School of Public Health, Indiana University, Bloomington, IN 47405, USA; tmickleb@iu.edu

**Keywords:** Pilates, core stability, pulmonary function, older adults, cardiorespiratory fitness, 6-minute walk test

## Abstract

**Background:** Identifying multimodal interventions to counteract age-related physiological decline is a critical public health priority. This study investigated the impact of an 8-week Mat Pilates intervention (MPT) on the interplay between core stability, pulmonary function, and cardiorespiratory fitness in older adults, specifically examining the mechanistic link between trunk stabilization and respiratory mechanics. **Methods:** Twenty older adults (18 females, 2 males; age 60—77 years) were randomized (stratified by sex, age, and baseline stability) into an MPT group (*n* = 10; 60-min sessions, 3×/week) or a control group (CON, *n* = 10). Primary outcomes included core stability (plank test), functional flexibility (sit-and-reach; back-scratch), pulmonary function (FVC, FEV_1_, FEV_1_/FVC, FEF_25–75%_, MVV), and cardiorespiratory fitness (6-min walk test; 6MWT). **Results:** Post-intervention, the MPT group demonstrated significant improvements in core stability, flexibility, and all pulmonary variables (FVC, FEV_1_, FEF_25–75%_, MVV) compared to the CON group (*p* < 0.001). A significant reduction in body weight was also observed (*p* < 0.001). Notably, MPT participants achieved superior 6MWT distances and reduced perceived exertion (*p* = 0.006). Correlation analysis revealed strong positive associations between core stability gains and pulmonary function (r = 0.892, *p* < 0.01), supporting the mechanistic link between trunk stabilization, enhanced ventilatory mechanics, and functional aerobic capacity. **Conclusions:** Mat Pilates is a potent intervention for older adults, facilitating a physiological synergy where core strengthening optimizes pulmonary function and cardiorespiratory endurance. These findings suggest MPT is a comprehensive modality for maintaining musculoskeletal and respiratory health, proving superior to habitual activity alone in promoting functional independence.

## 1. Introduction

The global population of older adults is expanding at an unprecedented rate. According to the United Nations, the number of individuals aged 60 and above is projected to reach 2.1 billion by the turn of the century [1,2]. This demographic shift presents a critical public health challenge, as biological aging is inherently characterized by a progressive decline in physical fitness. Reductions in musculoskeletal strength, postural control, and joint mobility often hinder the performance of daily activities, eventually compromising functional independence [3,4]. These declines are particularly aggressive in sedentary populations, where muscle strength may decrease by up to 14% per decade after the age of 50 [5].

However, the impact of aging extends beyond the musculoskeletal system. Age-related changes in spinal morphology, such as increased thoracic kyphosis, restrict chest wall expansion. When coupled with the weakening of primary respiratory muscles and diaphragmatic thinning, these alterations significantly impair pulmonary function and gas exchange efficiency [6,7]. This reduction in ventilatory capacity creates a “ceiling effect” on cardiorespiratory fitness, as the inability to meet oxygen demands during exertion leads to premature fatigue and a heightened risk of falls.

To mitigate these systemic declines, Pilates training has emerged as a promising mind–body intervention that bridges the gap between musculoskeletal strength and physiological efficiency. Grounded in the principles of centering, concentration, and controlled breathing, Pilates uniquely emphasizes the activation of the “powerhouse”, the deep stabilizing muscles of the core, including the transverse abdominis, multifidus, and pelvic floor [8]. While traditional resistance training often focuses on isolated muscle groups, Pilates promotes a mechanical synergy between respiratory mechanics and trunk stability.

This relationship is fundamentally functional. Research has demonstrated that Pilates not only improves functional capacity but also significantly enhances the thickness of the transverse abdominis and internal obliques [9]. These muscles serve a dual purpose: providing the postural support necessary for balance and acting as primary drivers for forced expiration. Supporting this, the deliberate breathing patterns in Pilates help maintain rib cage elasticity, while core stabilization directly influences diaphragmatic excursion, thereby improving ventilatory efficiency in aging populations [10,11].

Consequently, the potential for Pilates to improve aerobic capacity remains a subject of active investigation. While recent work confirmed that Pilates enhances oxygen utilization and metabolic efficiency [12], the direct mechanistic link between core-based breathing techniques and improved 6-min walk test (6MWT) performance remains under-investigated. This is a critical omission, as the 6MWT is not merely a measure of lower-limb strength, but a complex reflection of global cardiorespiratory coordination [13].

This study aims to bridge this gap by examining the synergy of these variables, testing the hypothesis that an 8-week Mat Pilates intervention facilitates a “co-activation” of the respiratory and musculoskeletal systems [14]. This perspective is reinforced by the suggestion that stabilizing the proximal kinetic chain is essential for reducing the energy cost of walking and improving systemic oxygen delivery [15]. Grounding our analysis in the observation that trunk stabilization is a prerequisite for optimizing peripheral oxygen delivery during gait [16], we aim to demonstrate that this integrated “core–pulmonary” efficiency drives superior gains in functional aerobic capacity [17,18].

Despite these documented benefits, there remains a paucity of literature directly comparing the impact of specific training programs on the intersection of core strength, pulmonary function, and cardiorespiratory fitness in older adults. Therefore, the purpose of this randomized controlled trial was to examine the impacts of an 8-week Mat Pilates program on core stability, pulmonary function, flexibility, and cardiorespiratory fitness in this population. We hypothesized that the intervention would yield significant improvements across all markers, with particularly pronounced gains in core stability and functional flexibility.

## 2. Materials and Methods

### 2.1. Study Design and Randomization

This randomized controlled trial (RCT) utilized a parallel-group design. An a priori sample size calculation using G*Power 3.1.9.7 (based on *d* = 0.89 [19], α = 0.05, power = 0.95) determined a minimum of 15 participants; 20 were enrolled to account for attrition. An independent researcher generated the random allocation sequence using block randomization (block size of 4), stratified by sex, age (60–69 and 70–79 years), and baseline core stability (plank test: 0–30 s or >30 s). Participants were allocated (1:1) into either Mat Pilates Training (MPT, *n* = 10) or Control (CON, *n* = 10) groups. To ensure allocation concealment, the sequence was held by a third party in sequentially numbered, opaque, sealed envelopes, opened only after baseline assessments. Due to the nature of exercise, participants and instructors were not blinded, but outcome assessors and data analysts remained blinded to group assignments throughout the study.

### 2.2. Participants and Ethical Considerations

Twenty healthy older adults (aged 60–77 years) were recruited from Nakhon Si Thammarat Province, Thailand. Eligible participants were non-smokers, capable of independent movement, and had not engaged in structured exercise or dietary supplementation for at least 6 months. Exclusion criteria comprised severe cardiovascular or respiratory diseases, acute musculoskeletal conditions (e.g., spondylolisthesis, scoliosis, or gout), and uncontrolled hypertension. To ensure participant safety, a Sports Medicine specialist supervised the entire intervention and data collection process. The study was conducted in accordance with the Declaration of Helsinki, and the protocol was approved by the Human Research Ethics Committee of Walailak University (No. WUEC-24-333-01) on 27 September 2024. All participants provided written informed consent following a comprehensive screening of medical and activity histories. The trial was prospectively registered with the Thai Clinical Trials Registry (TCTR20260304009) on 4 March 2026. Participant recruitment commenced on 30 September 2024, and all follow-up assessments were concluded by 15 March 2025

### 2.3. Assessment

#### 2.3.1. Body Composition and Vital Signs

Baseline hemodynamics, including resting heart rate (RHR) and blood pressure (BP), were measured using a digital sphygmomanometer (Microlife BP B1 Standard, Taipei, Taiwan) following a 10-min seated rest. Peripheral oxygen saturation (SpO_2_) was monitored via pulse oximetry (Finger Pulse Oximeter M70c, Guangdong, China). Body composition, specifically skeletal muscle mass and body fat percentage, was analyzed via bioelectrical impedance analysis (BIA) using the ACCUNIQ BC380 (Selvas Healthcare, Daejeon, Republic of Korea). To ensure data reliability, participants followed a standardized protocol: fasting for ≥ 3h and voiding the bladder immediately prior to testing.

#### 2.3.2. Core Stability Testing

Core endurance was evaluated using the prone bridge (plank) test. Participants were instructed to maintain a neutral spine position while supported by their forearms and toes. The test concluded when the participant could no longer hold the correct form, with the total time in seconds recorded as the measure of core stability [20].

#### 2.3.3. Pulmonary Function Testing

Pulmonary function was evaluated using a calibrated computerized spirometer (VO_2_max Tracker Ergospirometer, MES, Kraków, Poland) in accordance with American Thoracic Society guidelines. While seated and wearing a nose clip, participants performed three maneuvers to ensure reproducibility. Criteria for acceptance required the two highest values for FVC, FEV_1_, FEV_1_/FVC, FEF_25–75%_ within 0.15 L. MVV was subsequently measured, and the best maneuvers were utilized for analysis.

#### 2.3.4. Flexibility Testing

Flexibility was assessed via two metrics: (1) lower body flexibility through the sit-and-reach test (ACSM guidelines), and (2) upper body flexibility via the back-scratch test [21]. For the latter, the distance between the middle fingers was recorded, with positive values indicating overlap and negative values representing a gap.

#### 2.3.5. Cardiorespiratory Fitness Testing

Functional exercise capacity was assessed using the 6-Minute Walk Test (6MWT) on a 40-m rectangular course, following the American Thoracic Society (ATS) technical standards. A longer rectangular track was utilized to minimize frequent directional changes and deceleration, thereby optimizing the walking rhythm [12]. After a 10-min seated rest, participants were instructed to cover the maximum distance possible in six minutes at their own pace. Standardized encouragement was provided, and the Borg Rating of Perceived Exertion scale (Borg scale) was employed to evaluate baseline and post-exercise dyspnea and fatigue [12,22]. The total distance in meters was recorded as a proxy for cardiorespiratory endurance.

### 2.4. Intervention

Mat Pilates training (MPT) protocol: The MPT group participated in a structured 8-week intervention consisting of 60-min group sessions, three times weekly. To ensure protocol fidelity and safety, sessions were led by two sports scientists (1:10 instructor-to-student ratio). The initial three weeks served as a foundational phase under a certified Pilates instructor, focusing on lateral breathing mechanics and postural alignment.

Subsequent sessions, conducted at the Tha Sala Subdistrict Municipality Office, followed a standardized structure: a 10-min warm-up, 40 min of Mat Pilates—incorporating bodyweight-based multi-directional stepping, limb coordination, and trunk rotation—and a 10-min cool-down. Exercise intensity was strictly maintained at 55–70% of heart rate reserve (HRR), monitored every 10 min via pulse oximetry. Progression was standardized bi-weekly by increasing repetitions as the Rate of Perceived Exertion (RPE) decreased. Exercises were modified individually if discomfort was reported, and no supplemental home exercises were prescribed (Table 1).

Control group (CON) protocol: Participants in the CON group were instructed to maintain their pre-existing lifestyle and dietary habits throughout the study. They continued their habitual routine physical activities, including their baseline one-hour walking sessions, but were strictly directed not to initiate any new exercise modalities for the duration of the 8 weeks.

### 2.5. Statistical Analysis

Statistical analyses were performed using IBM SPSS Statistics (version 27.0; IBM Corp., Armonk, NY, USA). Normality of distribution was verified via the Shapiro–Wilk test. A two-way mixed-model ANOVA assessed the main effects of time, group, and their interaction across all variables. Significant interactions were followed by Bonferroni-adjusted post hoc pairwise comparisons. To evaluate the magnitude of the intervention effect, independent t-tests compared delta scores (Δ, post-test minus pre-test) between the MPT and CON groups. Pearson’s correlation coefficients (r) examined the relationships between delta changes (Δ) in core, pulmonary, and cardiorespiratory metrics. Results are presented as Mean ± Standard Deviation (SD) with 95% Confidence Intervals (CIs). The threshold for statistical significance was set at *p* < 0.05.

## 3. Results

**Figure 1:**
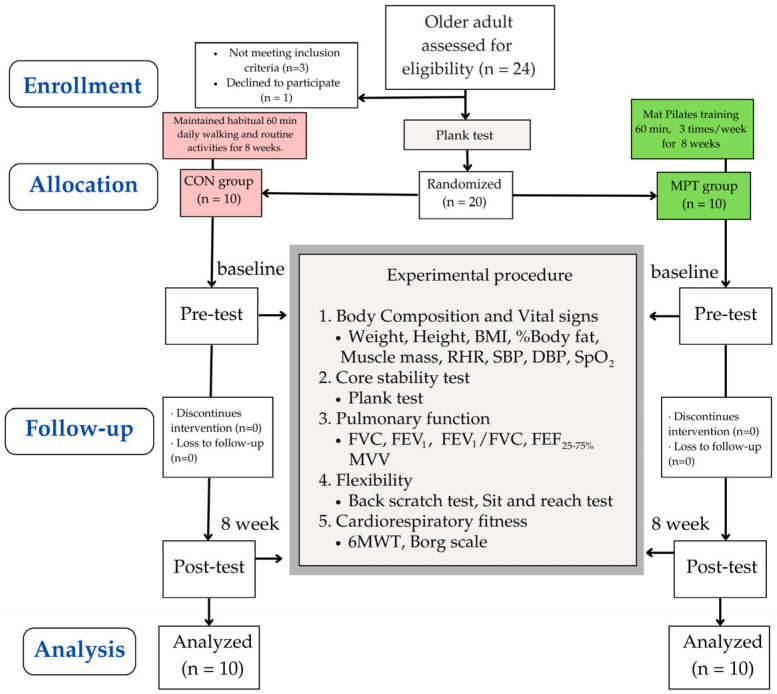
Experimental testing overview. The pink boxes indicate the CON group, while the green boxes represent the MPT group. Arrows describe the chronological flow of the experimental procedure.

A total of 20 older adults (18 females, 2 males) completed the 8-week study with full adherence and no reported adverse events or exercise-related injuries. Baseline characteristics, including age, sex, weight, and height, showed no significant differences between the Mat Pilates Training (MPT) and Control (CON) groups (*p* > 0.05), indicating a well-matched baseline (Figure 1).

### 3.1. Physiological and Anthropometric

Physiological and anthropometric changes are summarized in Table 2. A significant Time × Group interaction effect was observed for body weight (*p* < 0.001, $\eta_{p}^{2}$ = 0.44). Specifically, the MPT group demonstrated a significant reduction of 1.36 ± 0.85 kg in body weight, whereas the CON group showed a slight increase (mean change = 0.95 ± 1.40 kg) over the 8 weeks. The comparison of delta scores (Δ) confirmed a significant difference in weight loss between the groups (t = −4.45, *p* < 0.001). No significant changes or interaction effects were observed for height (*p* = 0.241). Notably, baseline characteristics, including sex and age distribution, were perfectly balanced between groups through stratified randomization.

### 3.2. Body Composition and Vital Signs

The 8-week intervention is summarized in Table 3. It did not result in significant Time × Group interaction effects for body composition markers, including BMI (*p* = 0.052, $\eta_{p}^{2}$ = 0.19), body fat percentage (*p* = 0.077, $\eta_{p}^{2}$ = 0.16), or muscle mass (*p* = 0.427, $\eta_{p}^{2}$ = 0.04). While the MPT group exhibited a downward trend in BMI compared to a marginal increase in the CON group, this between-group difference in delta scores was not statistically significant (t = 2.08, *p* = 0.052). However, the large effect size ($\eta_{p}^{2}$ = 0.19) suggests a clinically meaningful trend towards weight management in the MPT group.

Regarding resting cardiovascular and respiratory parameters, no significant interaction or main effects were observed for RHR, SBP, or DBP (*p* > 0.05). All vital signs remained within normal clinical ranges throughout the study. Similarly, changes in peripheral oxygen saturation (SpO_2_) did not differ significantly between the MPT (0.60 ± 0.97%) and CON (0.30 ± 0.82%) groups (*p* = 0.464, $\eta_{p}^{2}$ = 0.03). No adverse hemodynamic events or musculoskeletal injuries were reported during the intervention or assessment periods.

### 3.3. Core Stability

A significant Time × Group interaction effect was observed for core stability, as measured by the plank test (*p* < 0.001, $\eta_{p}^{2}$ = 0.85). The MPT group demonstrated a substantial increase in hold time compared to the CON group, which showed a slight decline. The comparison of delta scores confirmed a significantly superior improvement in the MPT group (t = −8.05, *p* < 0.001).

### 3.4. Pulmonary Function

Significant Time × Group interactions were found across all pulmonary function variables (*p* < 0.001). Specifically, FVC ($\eta_{p}^{2}$ = 0.86), FEV_1_ ($\eta_{p}^{2}$ = 0.80), FEV_1_/FVC ($\eta_{p}^{2}$ = 0.63), FEF_25–75%_ ($\eta_{p}^{2}$ = 0.75), and MVV ($\eta_{p}^{2}$ = 0.77) all showed significant improvements in the MPT group compared to the CON group. Between-group comparisons of delta scores corroborated these findings, with the largest group differences observed in FVC (t = −10.92, *p* < 0.001) and FEV_1_ (t = −8.80, *p* < 0.001).

### 3.5. Flexibility

Flexibility significantly improved following the 8-week MPT intervention. Interaction effects were significant for both the back-scratch test (*p* < 0.001, $\eta_{p}^{2}$ = 0.66) and the sit-and-reach test (*p* < 0.001, $\eta_{p}^{2}$ = 0.76). The MPT group exhibited a mean increase of 6.60 ± 2.91 cm in the sit-and-reach test, whereas the CON group showed a decrease (t = −7.57, *p* < 0.001).

### 3.6. Cardiorespiratory Fitness

Cardiorespiratory fitness outcomes are detailed in Table 4. A significant Time × Group interaction effect was observed for the 6MWT distance (*p* = 0.006, $\eta_{p}^{2}$ = 0.35). The MPT group exhibited a significant increase in walking distance (95.10 ± 80.98 m), whereas the CON group remained relatively stable. The difference in delta scores between groups was statistically significant (t = −3.09, *p* = 0.006).

Consistent with the 6MWT improvements, a significant interaction effect was observed for the Borg scale scores (*p* = 0.002, $\eta_{p}^{2}$ = 0.41). Post-intervention scores significantly decreased in the MPT group, while a slight increase was recorded in the CON group. Between-group analysis of delta scores confirmed the superior reduction in perceived exertion for the MPT group (t = 3.56, *p* = 0.002).

### 3.7. Correlation Analysis of Physiological Adaptations

**Figure 2:**
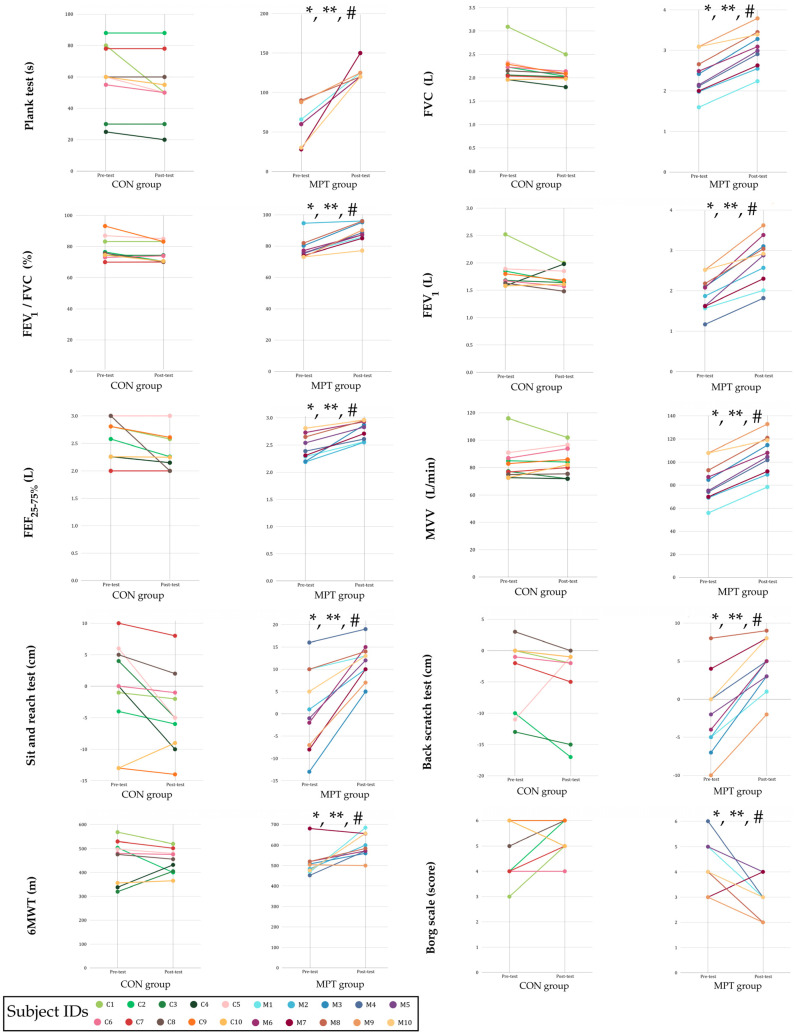
Individual changes in core stability, pulmonary function, flexibility, and cardiorespiratory fitness across the 8-week intervention. Left panels display the control group (CON, *n* = 10); right panels display the Mat Pilates training group (MPT, *n* = 10). Individual lines represent Subject IDs (C1—C10 and M1—M10). FVC, forced vital capacity; FEV_1_, forced expiratory volume in 1 s; FEV_1_/FVC, forced expiratory volume in 1 s/forced vital capacity ratio; FEF_25–75%_, forced expiratory flow between 25% and 75% of vital capacity; 6MWT, 6-min walk test. Data are presented as means ± standard deviations; * *p* < 0.05, significant within-group difference between pre- and post-test; ** *p* < 0.05, significant Time × Group interaction effect; # *p* < 0.05, significant difference in Delta Score (Δ) between groups.

Pearson’s analysis identified potent positive correlations between enhanced core stability and respiratory adaptations (Table 5). Notably, the change (Δ) in the plank test was strongly correlated with gains in pulmonary function, specifically FVC (r = 0.892), MVV (r = 0.847), and FEF_25–75%_ (r = 0.842, all *p* < 0.01). Furthermore, functional endurance (6MWT) exhibited moderate but significant correlations with core stability (*p* < 0.05). Interestingly, while flexibility (sit-and-reach) also demonstrated a significant positive relationship with 6MWT performance (r = 0.567, *p* < 0.01) (Figure 2).

## 4. Discussion

To our knowledge, this is the first study to comprehensively examine the interplay between core stability, pulmonary function, and cardiorespiratory fitness in an older cohort through a structured Mat Pilates (MPT) intervention. Our findings demonstrate that an 8-week regimen (60-min sessions, 3 times/week) yielded significant improvements across all measured physiological domains. Notably, although our cohort was predominantly female, the inclusion of male participants—balanced through stratified randomization—allowed for a broader assessment of Mat Pilates’ physiological impacts across the older adult population. This rigorous approach ensures that the observed improvements in core–pulmonary synergy are not merely gender-specific but represent a fundamental physiological adaptation to the MPT regimen. Consequently, these results position MPT as a potent multi-modal intervention for mitigating age-related declines in functional capacity.

The significant enhancement in core stability, evidenced by plank test performance, aligns with evidence that Pilates improves abdominal endurance and global strength [23]. Unlike isolated resistance training, the isometric and isokinetic nature of Pilates optimizes muscle thickness and postural balance [24,25]. We posit that the plank serves as a functional cornerstone; by stabilizing the proximal kinetic chain, it facilitates more efficient movement patterns and enhances overall physical resilience in older adults [26,27], as demonstrated by the consistent improvements observed across our cohort.

A pivotal finding was the significant improvement in pulmonary parameters, including FVC, FEV_1_, FEV_1_/FVC, FEF_25–75%_, and MVV. We attribute these gains to the “lateral breathing” principle, which emphasizes deep thoracic expansion and diaphragmatic control during exertion [26,28]. Our data reinforce the theory that a stable core provides a superior mechanical platform for respiratory muscle function [29,30]. Specifically, the correlation between abdominal strength and lung volumes suggests that MPT-induced trunk stabilization reduces chest wall impedance. These respiratory adaptations are consistent with recent literature observing improved ventilatory capacity across diverse aging populations [18,31]. The synergy between controlled breathing and resistance exerted a substantial effect, serving as a potent stimulus for mitigating physiological decline [32,33].

The MPT group exhibited marked improvements in back-scratch and sit-and-reach tests, suggesting that Pilates effectively addresses joint stiffness and reduces lower-body discomfort [34,35]. This is not merely an isolated gain in range of motion; improved flexibility in older adults is intrinsically linked to neuromuscular control and strength, which collectively facilitate safer and more efficient functional movement and daily autonomy [36,37].

Marked improvements in 6MWT performance post-intervention highlight the robust nature of MPT in enhancing aerobic capacity [38]. These gains mirror findings that Pilates can optimize cardiovascular parameters and gait even in clinical populations [39]. Crucially, the 6MWT improvements were strongly correlated with gains in pulmonary function and core stability, validating our hypothesis of an integrated core–respiratory adaptation. We propose that a stabilized core reduces the “oxygen cost of breathing,” allowing for better ventilatory mechanics and sustained cardiorespiratory effort during gait [36,40]. The combination of aerobic and resistance elements inherent in MPT thus drives a global enhancement of functional endurance [41,42].

Collectively, our results support the presence of a functional “core–pulmonary” axis, wherein improved core stabilization serves as a mechanical prerequisite for enhanced ventilatory efficiency and aerobic performance. The strong correlations observed between plank endurance and pulmonary metrics suggest that MPT does not merely improve muscle strength in isolation but likely optimizes the bellows-like action of the thoracic cage. This integrated adaptation potentially reduces the work of breathing during exertion, which explains the marked improvements in 6MWT performance observed across our diverse cohort. By addressing the interplay between musculoskeletal stability and respiratory mechanics, an 8-week MPT regimen appears to be a robust and viable strategy for improving global functional capacity in older adults. These findings emphasize that the synergy of mind–body movement and controlled breathing may be more effective for maintaining cardiorespiratory independence than traditional, single-domain exercise modalities, offering a scalable intervention for the aging population.

## 5. Limitations and Future Directions

Despite the significant findings, this study has limitations. Environmental variables, such as room humidity, were not strictly controlled. While our sample was predominantly female (90%), the inclusion of male participants—balanced through stratified randomization—represents an initial step toward understanding the intervention’s broader applicability in older adults. However, we acknowledge that the small number of male subjects precludes a robust sex-disaggregated analysis. Future research should investigate these mechanisms in larger, gender-balanced cohorts to further explore potential sex-based differences in respiratory–motor coupling during Pilates, as well as in clinical populations with chronic respiratory disease. Long-term follow-up studies comparing MPT directly with traditional aerobic exercise are also warranted to evaluate the sustainability of these physiological adaptations.

## 6. Conclusions

In conclusion, this study demonstrates that an 8-week Mat Pilates intervention is an effective multi-modal strategy for improving core stability, pulmonary function, and cardiorespiratory fitness in older adults, regardless of their baseline physical status. Our findings highlight a clear physiological interplay: enhanced core stability provides the mechanical foundation necessary for improved pulmonary function, which in turn facilitates superior functional endurance as measured by the 6MWT. These results suggest that Pilates is a highly effective intervention for older adults, emphasizing that exercise selection should be tailored to individual accessibility to ensure long-term adherence and systemic health optimization.

## Figures and Tables

**Table 1:** Detailed Mat Pilates Training Protocol.

Phase	Exercises	Dosage & Progression	Time (mins)
Warm up	Breathing, Imprint & Release, Hip Release, Hip Rolls, Scapula Protraction/Retraction, Arm Circles, Scapula Elevation/Depression, Head Nods, Spine Rotation, Clam Shells	5 reps per exercise. Focus on foundational breathing and ribcage placement.	10
Mat Pilates program (Main Phase)	Toe Taps, Ab Prep, Prone Heel Squeeze, Single Leg Extension, One Leg Circle, Shoulder Bridge Prep 1 & 2, Hundred Prep, Roll Up Prep, Side Leg Lifts, Breaststroke Preps, Breaststroke, Swimming, Half Roll Back, Birddog	5 reps per exercise (Weeks 1–3). Progression: Repetitions increased bi-weekly based on RPE and HRR (55–70%).	40
Stretching (Cool-down)	Spine Stretch Forward, Shell Stretch, Side Bending, Cat Stretch, Roll Down	5 reps per exercise. Focus on spinal mobility and heart rate recovery.	10

**Table 2:** Physiological and Stratification Characteristics.

Variables	Group	(*n*, %)		Baseline		*p*-Value (t)
Strata Factors						
Sex	CON (*n* = 10)	Male (1, 10%)	Female (9, 90%)			>0.99
	MPT (*n* = 10)	Male (1, 10%)	Female (9, 90%)			
Age group (years)	CON (*n* = 10)	60–69 years (6, 60%)	70–79 years (4, 40%)	68.90 ± 5.49		0.948 (−0.07)
	MPT (*n* = 10)	60–69 years (6, 60%)	70–79 years (4, 40%)	68.40 ± 4.32		
Core stability (Plank) (s)	CON (*n* = 10)	0–30 s (2, 20%)	>30 s (8, 80%)	59.60 ± 20.16		0.824 (0.23)
	MPT (*n* = 10)	0–30 s (2, 20%)	>30 s (8, 80%)	60.20 ± 20.12		
**Variables**	**Group**	**Pre-test**	**Post-test**	**Delta Score (Δ)**	***p*-value** **(** $\eta_{p}^{2}$ **)**	***p*-value** **(t)**
**Continuous**						
Weight (kg)	CON (*n* = 10)	57.87 ± 5.98	58.82 ± 5.64	0.95 ± 1.40	<0.001 ** (0.44)	<0.001 # (−4.45)
	MPT (*n* = 10)	57.80 ± 7.39	56.44 ± 7.71	−1.36 ± 0.85 *		
Height (cm)	CON (*n* = 10)	157.40 ± 3.44	157.50 ± 3.57	0.10 ± 0.74	0.241 (0.08)	0.243 (−1.21)
	MPT (*n* = 10)	155.40 ± 5.82	156.00 ± 5.93	0.60 ± 1.07		

Data are presented as means ± standard deviations; * *p* < 0.05, significant within-group difference between pre- and post-test; ** *p* < 0.05, significant Time × Group interaction effect; ^#^ *p* < 0.05, significant difference in Delta Score (Δ) between groups.

**Table 3:** Comparison of Body Composition and Vital Signs variables between the CON group and MPT groups at baseline and post-intervention.

Variables	Group	Pre-Test	Post-Test	Delta Score (Δ)	*p*-Value ($\eta_{p}^{2}$)	*p*-Value (t)
BMI (kg/m^2^)	CON (*n* = 10)	25.17 ± 3.99	25.40 ± 4.02	0.22 ± 0.66	0.052 (0.19)	0.052 (2.08)
	MPT (*n* = 10)	24.26 ± 4.81	23.61 ± 4.23	−0.65 ± 1.15		
Body Fat (%)	CON (*n* = 10)	34.26 ± 9.08	35.27 ± 8.89	1.01 ± 1.46	0.077 (0.16)	0.077 (1.88)
	MPT (*n* = 10)	33.68 ± 5.44	33.32 ± 5.51	−0.32 ± 1.70		
Muscle mass (kg)	CON (*n* = 10)	33.41 ± 2.54	33.56 ± 2.54	0.15 ± 0.25	0.427 (0.04)	0.427 (0.50)
	MPT (*n* = 10)	33.34 ± 3.34	33.38 ± 2.78	0.04 ± 0.35		
RHR (bpm)	CON (*n* = 10)	76.80 ± 9.83	77.00 ± 10.0	0.20 ± 3.79	0.500 (0.03)	0.500 (−0.69)
	MPT (*n* = 10)	76.20 ± 4.98	77.50 ± 3.89	1.30 ± 3.33		
SBP (mmHg)	CON (*n* = 10)	131.60 ± 7.00	131.90 ± 4.20	0.30 ± 7.29	0.683 (0.01)	0.683 (0.42)
	MPT (*n* = 10)	132.80 ± 3.91	132.00 ± 6.65	−0.80 ± 4.16		
DBP (mmHg)	CON (*n* = 10)	73.80 ± 11.25	72.30 ± 7.23	−1.50 ± 5.95	0.895 (0.00)	0.895 (−0.13)
	MPT (*n* = 10)	78.00 ± 12.28	76.80 ± 10.71	−1.20 ± 3.82		
SpO_2_ (%)	CON (*n* = 10)	96.90 ± 0.74	97.20 ± 0.63	0.30 ± 0.82	0.464 (0.03)	0.464 (−0.75)
	MPT (*n* = 10)	97.40 ± 0.97	98.00 ± 0.82	0.60 ± 0.97		

BMI, body mass index; RHR, resting heart rate; SBP, systolic blood pressure; DBP, diastolic blood pressure; SpO_2_, peripheral oxygen saturation. Data are presented as means ± standard deviations.

**Table 4:** Comparison of Core Stability, Pulmonary Function, Flexibility, and Cardiorespiratory Fitness between the CON group and MPT groups at baseline and post-Intervention.

Variables	Group	Pre-Test	Post-Test	Delta Score (Δ)	*p*-Value ($\eta_{p}^{2}$)	*p*-Value (t)
Plank test (s)	CON (*n* = 10)	59.60 ± 20.16	53.60 ± 19.37	−6.00 ± 9.07	<0.001 ** (0.85)	<0.001 # (−8.05)
	MPT (*n* = 10)	60.20 ± 20.12	124.00 ± 9.37	63.80 ± 25.88 *		
FVC (L)	CON (*n* = 10)	2.27 ± 0.31	2.10 ± 0.19	−0.16 ± 0.18	<0.001 ** (0.86)	<0.001 # (−10.92)
	MPT (*n* = 10)	2.29 ± 0.41	2.95 ± 0.47	0.67 ± 0.17 *		
FEV1(L)	CON (*n* = 10)	1.84 ± 0.27	1.70 ± 0.19	−0.14 ± 0.15	<0.001 ** (0.80)	<0.001 # (−8.80)
	MPT (*n* = 10)	1.86 ± 0.38	2.69 ± 0.59	0.83 ± 0.31 *		
FEV1/FVC (%)	CON (*n* = 10)	78.12 ± 7.30	75.46 ± 6.00	−3.06 ± 3.51	<0.001 ** (0.63)	<0.001 # (−5.40)
	MPT (*n* = 10)	78.63 ± 6.25	89.76 ± 6.92	10.69 ± 7.25 *		
FEF 25−75% (L)	CON (*n* = 10)	2.50 ± 0.26	2.30 ± 0.22	−0.18 ± 0.17	<0.001 ** (0.75)	<0.001 # (−6.68)
	MPT (*n* = 10)	2.46 ± 0.22	2.76 ± 0.16	0.30 ± 0.15 *		
MVV (L/min)	CON (*n* = 10)	83.63 ± 12.97	85.62 ± 9.50	2.00 ± 6.60	<0.001 ** (0.77)	<0.001 # (−7.73)
	MPT (*n* = 10)	82.60 ± 17.03	106.12 ± 16.42	23.52 ± 5.82 *		
Back−scratch test (cm)	CON (*n* = 10)	−0.60 ± 7.66	−4.20 ± 6.32	−3.60 ± 4.81	<0.001 ** (0.66)	<0.001 # (−5.88)
	MPT (*n* = 10)	1.10 ± 9.17	11.80 ± 4.71	10.70 ± 6.00 *		
Sit & Reach test (cm)	CON (*n* = 10)	−2.20 ± 5.12	−6.50 ± 6.08	−4.30 ± 3.50	<0.001 ** (0.76)	<0.001 # (−7.57)
	MPT (*n* = 10)	−2.10 ± 5.32	4.50 ± 3.41	6.60 ± 2.91 *		
6MWT (m)	CON (*n* = 10)	442.60 ± 90.49	440.16 ± 55.24	−2.44 ± 58.55	0.006 ** (0.35)	0.006 # (−3.09)
	MPT (*n* = 10)	508.80 ± 64.32	603.90 ± 57.37	95.10 ± 80.98 *		
Borg scale (score)	CON (*n* = 10)	4.80 ± 1.14	5.20 ± 0.79	0.40 ± 1.07	0.002 ** (0.41)	0.002 # (3.56)
	MPT (*n* = 10)	4.30 ± 0.95	3.00 ± 0.82	−1.30 ± 1.06 *		

FVC, forced vital capacity; FEV1, forced expiratory volume in 1 s; FEV1/FVC, forced expiratory volume in 1 s/forced vital capacity ratio; FEF25–75%, forced expiratory flow between 25% and 75% of vital capacity; 6MWT, 6-min walk test. Data are presented as means ± standard deviations; * *p* < 0.05, significant within-group difference between pre- and post-test; ** *p* < 0.05, significant Time × Group interaction effect. # *p* < 0.05, significant difference in Delta Score (Δ) between groups.

**Table 5:** Pearson’s correlation coefficients for the relationships between changes (Δ) in core stability, pulmonary function, flexibility, and cardiorespiratory fitness across both groups.

Variables	Plank Test	FVC	FEV_1_	FEV_1_/FVC	FEF_25–75%_	MVV	Back Scratch Test	Sit and Reach Test	6MWT
Plank test	1.000								
FVC	0.892 **	1.000							
FEV_1_	0.834 **	0.932 **	1.000						
FEV_1_/FVC	0.662 **	0.814 **	0.791 **	1.000					
FEF_25–75%_	0.842 **	0.885 **	0.812 **	0.695 **	1.000				
MVV	0.847 **	0.975 **	0.919 **	0.798 **	0.890 **	1.000			
Back scratch test	0.826 **	0.794 **	0.840 **	0.689 **	0.721 **	0.745 **	1.000		
Sit and reach test	0.819 **	0.783 **	0.802 *	0.658 *	0.779 **	0.747 **	0.804 **		
6MWT	0.547 **	0.556 *	0.464 *	0.297	0.548 *	0.527 *	0.193	0.567 **	1.000

* Correlation is significant at *p* < 0.05. ** Correlation is significant at *p* < 0.01; FVC, forced vital capacity; FEV_1,_ forced expiratory volume in 1 s; FEV_1_/FVC, forced expiratory volume in 1 s/forced vital capacity ratio; FEF_25–75%,_ forced expiratory flow between 25% and 75% of vital capacity; MVV, Maximum Voluntary Ventilation; 6MWT, 6-min walk test. Data are presented as Pearson’s correlation coefficients (r values).

## Data Availability

The datasets generated and analyzed for this study are available from the corresponding author upon reasonable request.
